# Effects of Insect-Resistant Maize 2A-7 Expressing mCry1Ab and mCry2Ab on the Soil Ecosystem

**DOI:** 10.3390/plants11172218

**Published:** 2022-08-26

**Authors:** Shuke Yang, Xin Liu, Xiaohui Xu, Hongwei Sun, Fan Li, Chaofeng Hao, Xingbo Lu

**Affiliations:** 1 Shandong Key Laboratory of Plant Virology, Institute of Plant Protection, Shandong Academy of Agricultural Sciences, Jinan 250100, China; 2 College of Life Sciences, Shandong Normal University, Jinan 250014, China

**Keywords:** transgenic insect-resistant maize 2A-7, soil ecosystem, physical and chemical properties, enzyme activity, microbial functional diversity

## Abstract

Transgenic maize 2A-7 expressing mCry1Ab and mCry2Ab has excellent resistance to lepidopteran pests. Previous studies have investigated the effects of several *Bacillus thuringiensis* (Bt) proteins on the soil. However, the effects of artificially modified Bt proteins on soil ecosystems are still unclear. To evaluate the effects of transgenic maize 2A-7 on soil, the physicochemical properties, enzyme activities and functional diversities of the microbial communities in rhizosphere soils from 2A-7 and its near-isogenic non-transgenic control Dongdan 6531 were analyzed at different developmental stages under field conditions. The alteration of six physicochemical properties (pH, total nitrogen, total phosphorus, organic matter, available phosphorus and alkali-hydrolyzed nitrogen) and six functional enzymes (catalase, alkaline phosphatase, sucrase, acid phosphatase, urease and alkaline protease) activities in the rhizosphere soils between the two maize cultivars were drastically correlated with plant growth stage, but not affected by the artificially modified Bt transgenes. An analysis of time-course Biolog data revealed that the functional diversity of microbial communities in the rhizosphere soil of 2A-7 and its control were similar at each developmental stage. The results suggest that transgenic maize 2A-7 has no significant impact on the soil ecosystem and provide valuable information on scientific safety assessments of 2A-7 and its commercial applications.

## 1. Introduction

The global area of genetically modified (GM) crops has increased year by year since its first commercial release in the United States in 1996 [1,2]. In 2019, over 16 million farmers in 29 countries grew GM crops commercially, and the global area of GM crops reached 190.4 million hectares, over 112 times more than that of 1996 [3]. China is not only the seventh largest producer of GM crops, but also the second largest consumer of GM crops [3]. In China, approximately 7–20% maize yield loss is caused by insect pests every year. The application of insect-resistant GM crops can effectively reduce the threat from various insects, including members of Lepidoptera, Diptera, Coleoptera and Hymenoptera. Importantly, the use of insect-resistant plants has greatly reduced the application of pesticides, which has brought significant environmental, social, and economic benefits [4].

The acquisition of insect-resistant trait in GM crops is due to the expression of a class of insecticidal crystal proteins derived from the Gram-positive bacterium *Bacillus* *Thuringiensis* (Bt) [5,6,7]. These proteins are broken down into small active toxin fragments in the target organism that can bind to the organism’s intestinal epithelial cells and cause perforations, thereby affecting the osmotic balance, eventually resulting in the death of the target organism [8,9,10]. The large-scale planting of GM crops worldwide has an impact on the ecological environment of the soil over the past two decades [11,12]. In general, Bt crops can accumulate Bt proteins in various tissues and organs, such as the leaf, stem, root and seed. Bt proteins can be released into soils in two ways: directly through plant rhizosphere exudates and through plant residues after GM crop-derived straw is returned to the soil [13,14]. Irrespective of how Bt proteins enter the soil, they can directly or indirectly cause changes in soil physical and chemical properties, affect soil enzyme activities and change the levels of soil microbial functional diversity and bacterial dominant flora [15,16]. Varying levels of Bt proteins have been detected in the rhizospheres of Bt cotton cultivars NuCOTN99B and SGK321 during their whole growth stages, with the highest concentrations reaching 200 ng/g and 300 ng/g, respectively [17]. In the rhizosphere soil of insect-resistant maize Mon810, the Cry1Ab protein content can reach 165 g/hectare [18]. In addition, with the application of GM crop-derived straw during cultivation, Bt proteins can enter the soil from the straw residue, which may have impacts on the soil ecological environments. The Bt proteins in root exudates of GM crops have been associated with clay minerals and humic acids of soil substrates, leading to retained insecticidal activity in the soil [19]. Additionally, the Cry1Ab protein in transgenic maize Mon810 accumulates in the rhizosphere soil at different plant developmental stages, and after the crop-derived straw is returned to the field, the degradation of the Bt proteins has two patterns: most Bt proteins degrade rapidly during the early stage, but a few Bt proteins degrade stably during the later stage [20].

Insect-resistant transgenic maize 2A-7, which expresses artificially modified codons of two Bt proteins Cry1Ab and Cry2Ab, was developed to control lepidopteran pests. Insect-resistant assays performed in both the field and laboratory revealed that 2A-7 is highly resistant to lepidopteran pests, such as the corn borer, armyworm and bollworm, and it also shows good resistance to *Spodoptera* *frugiperda* [21]. A recent study showed that the application of 2A-7 had no adverse effect on the non-target arthropod communities [22]. Therefore, 2A-7 is a candidate with good application prospects. The modifications of the two Bt proteins in 2A-7 may produce distinct characteristics, including expression patterns, protein stability levels and degradation patterns. Although numerous studies have investigated the effects of GM crops expressing the Cry1Ab or Cry2Ab protein on the soil [23,24,25], none have reported the effects of GM crops containing modified Cry1Ab and Cry2Ab proteins on the soil ecosystem.

By comparing the physical and chemical properties, enzyme activities and microbial diversity levels between the rhizosphere soil of the GM maize and that of its corresponding non-transgenic control in the field under natural conditions at different growth stages, the impact of the GM maize on the soil ecological environment can be assessed [26,27,28]. Thus, in this paper, the effects of transgenic maize 2A-7 on the soil environment were systematically evaluated. Our results provide scientific data related to the evaluation of the environmental safety of 2A-7 and establish a basis for government decision making of 2A-7 commercialization.

## 2. Results

### 2.1. Effects of Transgenic Insect-Resistant Maize 2A-7 on the Physical and Chemical Properties of Rhizosphere Soil

In this study, the pH, total nitrogen, total phosphorus, organic matter, available phosphorus and alkaline-hydrolyzed nitrogen of the rhizosphere soils of transgenic maize 2A-7 and its non-transgenic control Dongdan 6531 were measured at five different developmental stages: the seedling, jointing, tasseling, silking and full ripe stages. The tested indices in the rhizosphere soils of the two maize inbred lines were similar at the same developmental stages (Table 1). Specifically, during the growth and development of both maize lines, the pH values of the rhizosphere soils showed gradual upward trends, from acidic (pH between 6.7 and 6.8) to alkaline (pH between 7.7 and 7.8). The total nitrogen contents were distributed between 0.12% and 0.15%, showing sharp increases during the seedling stages, followed by slightly decreases during the other stages. The total phosphorus contents remained stable among the five stages, with distributions between 0.08% and 0.12%. The organic matter contents ranged from 17 g/kg to 27 g/kg during the five different developmental stages, but there was no significant difference between the two maize lines at each of the developmental stages. The available phosphorus contents in the rhizosphere soils of the two maize inbred lines gradually decreased as the development process advanced, ranging from 57.27 mg/kg to 98.63 mg/kg. The alkali-hydrolyzed nitrogen contents were distributed between 75.47 mg/kg and 91.27 mg/kg, and they remained stable during each period. Based on the above results, the maize developmental period, but not the transgenic event, appeared to be a vital factor affecting the physical and chemical properties of the rhizosphere soil.

### 2.2. Effects of Transgenic Insect-Resistant Maize 2A-7 on the Enzyme Activity Levels in Rhizosphere Soil

**Figure 1:**
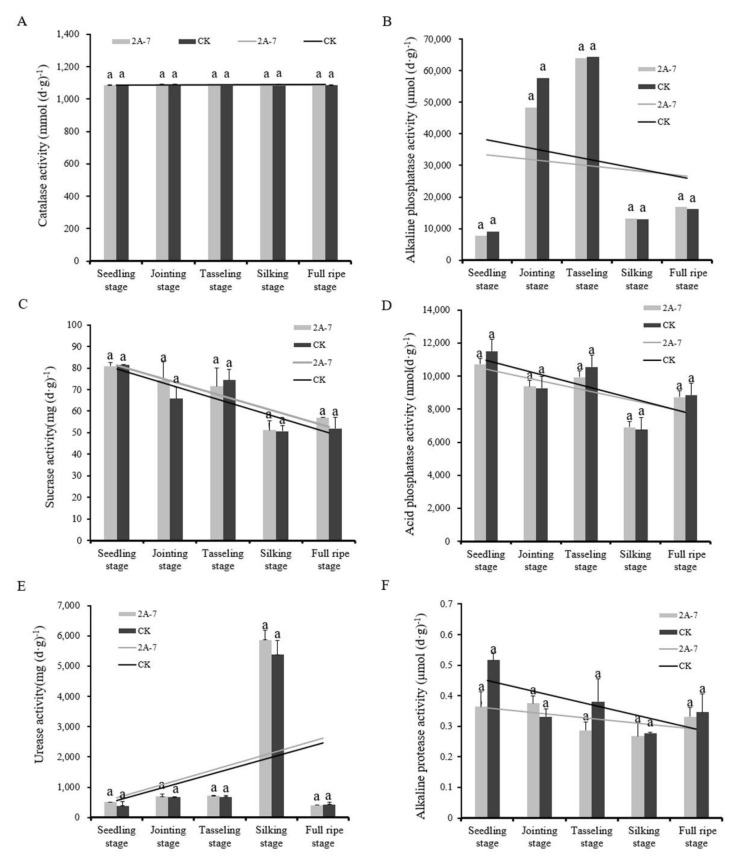
Effects of transgenic maize 2A-7 on the enzyme activities in the rhizosphere soil at different developmental stages. (**A**) Catalase activity; (**B**) alkaline phosphatase activity; (**C**) sucrase activity; (**D**) acid protease activity; (**E**) urease activity; (**F**) alkaline protease activity. 2A-7 represents the transgenic maize 2A-7, and CK represents the non-transgenic control Dongdan 6531. Letters above the columns indicate significant differences in rhizosphere soil treatments between the two maize lines at the same developmental stage (*p* < 0.05). The linear trend lines were graphed for the two maize cultivars at the five developmental stages.

The activities of six functional soil enzymes in the rhizosphere soil of transgenic maize 2A-7 and its non-transgenic control Dongdan 6531 at different developmental stages were analyzed. As shown in Figure 1, the activity levels of each enzyme were not significantly different in the rhizosphere soils of the two maize inbred lines during the same growth stage. During the five developmental periods, the catalase activities in the 2A-7 and the control maize rhizosphere soils were relatively stable (Figure 1A). The alkaline phosphatase activity showed a gradual upward trend during the first three stages and then decreased in the latter two stages (Figure 1B). The sucrase and acid phosphatase activities in the rhizosphere soils of the two maize inbred lines had similar trends throughout the maize growth period, showing gradual decreases from the seedling to silking stages, followed by slight increases (Figure 1C,D). The urease activities in the rhizosphere soils of the two maize inbred lines peaked during the silking stage, and the activity levels were similar for the rest of the developmental stages (Figure 1E). The alkaline protease activities in the rhizosphere soils of the two maize inbred lines were not significantly different at each developmental stage (Figure 1F). Thus, the transgenic maize 2A-7 did not appear to have a significant impact on the soil enzymes, whereas the growth stages appeared to have been highly influential.

### 2.3. Metabolic Functional Diversity of Microorganisms in the Rhizosphere Soils of Transgenic Maize 2A-7 and Its Control

#### 2.3.1. Metabolic Activity Changes in Microorganisms Inhabiting the Rhizosphere Soils of Transgenic Maize 2A-7 and ITS Control

**Figure 2:**
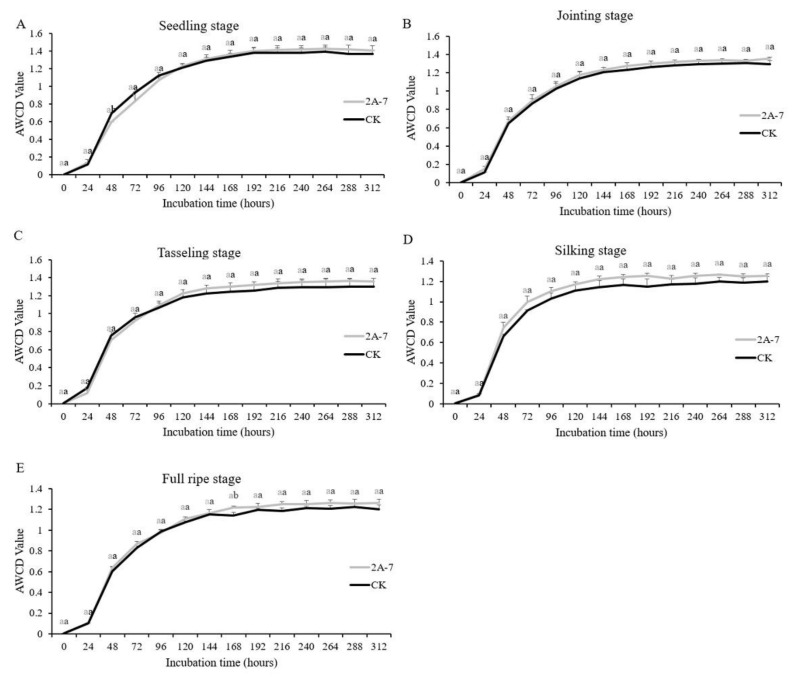
Changes in the microbial average well color development (AWCD) values of the rhizosphere soils of transgenic maize 2A-7 and its non-transgenic control during different developmental periods. (**A**) Changes of AWCD values at the seedling stage; (**B**) Changes of AWCD values at the jointing stage; (**C**) Changes of AWCD values at the tasseling stage; (**D**) Changes of AWCD values at the silking stage; (**E**) Changes of AWCD values at the full ripe stage. 2A-7 represents the transgenic maize 2A-7, and CK represents the non-transgenic control Dongdan 6531. Letters above the curves indicate significant differences in rhizosphere soil treatments between the two maize lines at the same developmental stage (*p* < 0.05).

Changes in values of the average well color development (AWCD) may reflect changes in the metabolic activity of a microbial community. More species and a greater abundance of soil microflora lead to more types and greater amounts of carbon sources that can be utilized in the plate cells. Generally speaking, the AWCD value is positively correlated with the growth of the cultured microbes. As shown in Figure 2, the changes in the AWCD values during different developmental stages showed a similar pattern, which was a linear increase during the early incubation period followed by a stationary stage during the latter periods. In each growth period, the AWCD values of the soil microorganisms of the two maize inbred lines increased slowly in the first 24 h, followed by rapid growth during the next 72 h, indicating that the microbes had high metabolic activities. After 96 h of culture, the increase rates of the AWCD values slowed and then plateaued. The AWCD curves of the rhizosphere soil microorganisms of the two maize inbred lines showed good coincidence, except during the silking stage. The results indicated that no significant differences exist in the microbial metabolic activities in the rhizosphere soils of the two maize inbred lines during the seedling, jointing, tasseling and full ripe stages.

#### 2.3.2. Changes in the Rhizosphere Soil Microorganisms’ Utilization of Different Carbon Sources between the Two Maize Lines

**Figure 3:**
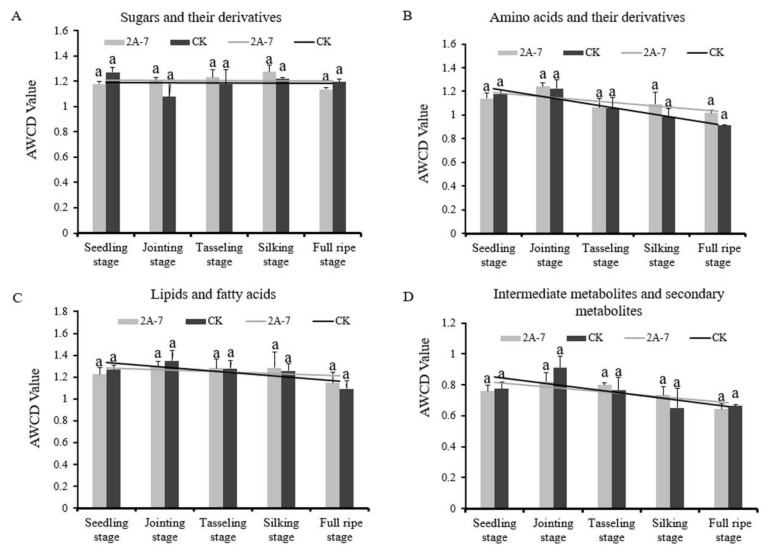
The utilization levels of four types of carbon sources by rhizosphere soil microorganisms of transgenic maize 2A-7 and its non-transgenic control during different developmental periods. (**A**) Sugars and their derivatives; (**B**) amino acid and their derivatives; (**C**) lipids and fatty acids; (**D**) intermediate metabolites and secondary metabolites. 2A-7 represents the transgenic maize 2A-7, and CK represents the non-transgenic control Dongdan 6531. Letters above columns indicate significant differences in rhizosphere soil treatments between the two maize lines at the same time node (*p* < 0.05). The linear trend lines were graphed for the two maize cultivars at the five developmental stages.

After 96h incubation, the microfloral utilization rate of the carbon sources slowed and then remained relatively steady (Figure 1). Thus, the carbon source utilization at this time point may reflect the differences in the utilization of individual carbon sources by microbes. From data collected after a 96 h incubation, the AWCD value of each type of carbon source was calculated. As shown in Figure 3, there were no significant differences in the utilization levels of the four types of carbon sources between the rhizosphere soil microorganisms of the two maize inbred lines at each developmental stage (Figure 3). The soil microbes from both types of maize utilized carbon source carbohydrates and their derivatives at a relatively stable state at the different developmental stages (Figure 3A), whereas the utilization of the other three types of carbon sources showed trends of first increasing and then decreasing, with the highest utilization rate at the jointing stage (Figure 3B–D). 

#### 2.3.3. Diversity Index Analysis of the Rhizosphere Soil Microbial Communities of Transgenic Maize 2A-7 and Its Control

The Shannon index (H′), Simpson index (D) and McIntosh index (U) indices of the rhizosphere soil microorganisms from the two maize lines during each developmental period were calculated using the 96 h soil microorganism carbon source utilization data. As shown in Table 2, at all the tested periods, the three diversity indices showed no significant differences between the microbial communities of the two maize lines. Therefore, we inferred that the transgenic maize 2A-7 had no significant impact on the species’ richness, dominance or homogeneity of the rhizosphere soil microorganisms.

## 3. Discussion

### 3.1. Effects of Transgenic Maize 2A-7 on Physical and Chemical Properties of Rhizosphere Soil

The physical and chemical properties of soil are important factors affecting soil quality and are used as important indicators for evaluating the level of soil fertility [29,30]. However, with the expansion of GM crop planting areas, more people have become worried about whether these crops pose a threat to the soil ecological environment. Exogenous proteins secreted from their roots or straw residue may change the physical and chemical properties of the soil, thereby affecting crop growth [31]. Changes in total nitrogen, total phosphorus and pH values in soil are not provoked by the planting of transgenic Bt maize, but they do occur with the return of GM crop-derived straw to the field [32]. In addition, the diversity of communities of Arbuscular Mycorrhizal Fungi in the soil is not changed by Bt maize [33]. Consistently, the planting of Bt cotton does not change the contents of total nitrogen, available phosphorus and available potassium in the soil, nor does it affect soil characteristics [34,35]. Here, our data provide evidence that the physical and chemical properties of the rhizosphere soil can be affected by the stages of maize development, but not affected by the transgenic event of Bt (Table 1). These results suggest that the planting of transgenic maize 2A-7 has no significant effects on the physical and chemical properties of the rhizosphere soil.

### 3.2. Effects of Transgenic Maize 2A-7 on Soil Enzyme Activities in Rhizosphere

Soil enzymes mainly originate from soil microorganisms, plant root exudates and the decomposition of animal and plant residues. Enzymes, such as phosphatase, urease, protease, invertase, cellulose and amylase, are key components in various biochemical reactions in soil, including the biotransformation of nitrogen, phosphorus, organic matter and carbon in soil, and their activities can be used to evaluate indicators of soil biotransformation direction and strength [36,37,38]. Enzyme activity levels are affected by environment, climate and planting conditions, such as temperature and humidity [39]. In general, soil enzyme activity is positively correlated with soil fertility. Transgenic maize and wheat do not affect the activities of urease, dehydrogenase and sucrase in rhizosphere soil at different developmental stages [40,41,42]. In this study, soil enzyme levels of one oxidoreductase and five hydrolases in the rhizosphere soils of transgenic maize 2A-7 and its control were determined. The activities of all six major soil functional enzymes did not significantly differ between the rhizosphere soils of the two maize lines during each developmental period (Figure 1). This result indicated that the changes in soil enzyme activities may be related to the maize developmental period.

### 3.3. Effects of Transgenic Maize 2A-7 on the Functional Diversity of Rhizosphere Soil Microorganisms

Microbes are involved in metabolic activities, such as the use of carbon and nitrogen sources, and microbial functional diversity is one of the important characteristics of biological communities in the soil ecological environment [43]. At present, GM crops have not been found to have caused significant impacts on soil microbial diversity [44,45]. The change trend of the AWCD value between phosphorus-efficient transgenic rice and its control is consistent, and the change in the microbial diversity index has only been related to fertilizer application and growth stages [46]. The rhizosphere microbial diversity index and a principal component analysis of transgenic maize Shuangkang 12-5 showed that the diversity level of its rhizosphere soil microorganisms is similar to that of its control maize [47]. The effects of transgenic insect-resistant maize expressing Crylle on the functional diversity levels of microbial communities in rhizosphere soils are not significantly different to those of its control maize Zong31, but the environmental conditions and plant growth stage appear to have stronger effects than the cultivar [48]. In this study, the changes in the AWCD values of the transgenic maize 2A-7 and its control Dongdan 6531 were similar. In the early stage of incubation (0–24 h), at all the tested periods, the AWCD values of the two maize inbred lines were low, close to 0, indicating that the metabolic activities of microbial communities in the rhizosphere soil of the transgenic maize 2A-7 and its control were low, with little of the carbon source being used [49,50,51]. After a 24 h incubation, the increase in the AWCD rate accelerated, indicating that the microbial community metabolic capacity increased, and the soil microorganisms gradually used a large amount of the carbon source. At 96 h, the AWCD curve’s slope for each treatment was the largest, indicating that the metabolic activity of the soil microbial community reached the highest level. From 144 h to 312 h, the increase in the AWCD rate gradually slowed and remained at a steady level, indicating that, due to factors such as competition and the limitation of a single carbon source, the increase in the number of microorganisms gradually decreased (Figure 2). Using the data collected after a 96 h incubation, the carbon source utilization capacity and three microbial diversity indices were analyzed. The utilization levels of the four types of carbon sources by the two maize lines were similar (Figure 3). The utilization of lipids and their derivatives, amino acids and their derivatives, intermediate metabolites and secondary metabolites was the highest at the jointing stage and then decreased (Figure 3). This result indicated that the rhizophere soil microbe communities of both the transgenic maize 2A-7 and its control require greater carbon sources during the vegetative stage compared with the reproductive stage. In addition, the utilization levels of carbohydrates and their derivatives remained stable throughout the whole growth period (Figure 3), implying the carbohydrates and their derivatives, as carbon sources, are necessary for the microbe communities. Three microbial diversity indices, H′, D and U, were not significantly different between transgenic 2A-7 and the non-transgenic control (Table 2). This result indicates that the species’ richness, dominance and homogeneity of the rhizosphere soil microbe communities were not affected by the transgenic maize 2A-7.

## 4. Materials and Methods

### 4.1. Plant Materials

Insect-resistant maize 2A-7 with Dongdan 6531 background and its control Dongdan 6531 were provided by China Agricultural University. The transgenic materials were first obtained by using the homozygous transgenic line 2A-7 with B73 background as the male parent, and 83B28 as the recurrent female parent. After successive backcrossing for 5 generations and then self-crossing for 2 generations, the homozygous transgenic line 2A-7 with 83B28 background was obtained. Thereafter, the hybrids containing 2A-7 event were obtained by crossing the 83B28 (2A-7) transgenic lines and PH6WC. The control maize lines Dongdan 6531 were the F1 hybrid of 83B28 and PH6WC.

### 4.2. Test Design

The transgenic maize 2A-7 and its non-transgenic control Dongdan 6531 were planted using a random block design, with three replicates. Each plot covered an area of 150 m^2^, with a row spacing of 60 cm and a plant spacing of 25 cm. There was a 1.0 m wide isolation belt between treatments, and the fields were under routine management. A five-point sampling method was used in this study, the whole root system of three plants at each point were dug out to collect rhizosphere soil samples in each plot. The rhizosphere soil samples were collected using the shake method [52] at five different developmental stages: the seedling (V3-V4 stage, the third leaf exposes 3 cm from the heart), jointing (V7-V8 stage, the total length of the stem nodes is 2–3 cm), tasseling (Vn stage, the tip of the plant tassel exposes 3–5 cm of the parietal leaves), silking (R1 stage, the filaments of the female ears protrude about 2 cm from the bracts) and full ripe (R6 stage, dry and hard kernels) stages. After filtering through 40-mesh sieves to remove impurities, the pure soils from one plot were mixed together as a biological replicate. The soil samples were put into Ziplock bags, transferred to the laboratory with ice bags, and then each sample was divided into two parts: one (≥120 g dry soils per sample) was air-dried indoors for the soil physicochemical properties determination, and the other part (≥15 g soil per sample) was activated immediately at room temperature for 24 h for a microbial functional diversity test.

### 4.3. Determination of Physical and Chemical Properties of the Rhizosphere Soil

The soil pH value was measured using the potentiometric method [53]. The contents of soil total nitrogen, total phosphorus, organic matter, available phosphorus and alkali-hydrolyzed nitrogen were measured using the methods described in a previous study [54].

### 4.4. Determination of Enzyme Activities in the Rhizosphere Soil

The soil enzyme activity levels were tested in accordance with the producer manual instructions. The soil catalase activities were determined with soil catalase (S-CAT) activity detection kits (BC0105, Solerbio, Beijing, China). The soil urease activities were determined with soil urease (S-UE) activity detection kits (BC0125, Solerbio, Beijing, China). The soil sucrase activities (S-SC) were determined with soil sucrase (S-SC) activity detection kits (BC0245, Solerbio, Beijing, China). The soil alkaline phosphatase activities (S-AKP/ALP) were determined with soil alkaline phosphatase (S-AKP/ALP) activity detection kits (BC0285, Solerbio, Beijing, China). The soil acid phosphate activities (S-ACP) were determined with soil acid phosphatase (S-ACP) activity detection kits (BC0140, Solerbio, Beijing, China). The soil alkaline protease activities were determined with soil alkaline protease (S-ALPT) activity detection kits (BC0885, Solerbio, Beijing, China).

### 4.5. Determination of Microbial Functional Diversity in the Rhizosphere Soil

A Biolog Eco plate was used for the microbial functional diversity determination, and the Eco plate inoculum was prepared using the centrifugal decarbonization method [55]. The AWCD value of each well was calculated to reflect the overall metabolic activity of soil microorganisms to 31 carbon sources using the method described in a previous study [56]. The diversity index analysis of the soil microbial community was calculated after a 96 h incubation. Three diversity indices, Shannon index (H′), Simpson index (D) and McIntosh index (U), were used to evaluate the species richness, dominance and homogeneity, respectively. The calculation formulae were as follows:

H = −∑Pi(lnPi)(1)

D = 1 − ∑(Pi)^2^ (2)

U = (∑(Ni)^2^)^½^ (3)

### 4.6. Data Processing

Data processing was performed using Microsoft Excel 2019 for data organization and graphing, and a GraphPad Prism (version 6.0) was used for the Student’s *t*-test analyses.

## 5. Conclusions

This study provided comprehensive information on the effects of transgenic maize 2A-7 on the soil ecosystem. All the data implied that the changes in the physicochemical properties, enzyme activities and microbial diversities are directly related to the growth period but not to the presence of exogenous genes. Therefore, to evaluate whether a GM crop has impacts on its rhizosphere soil ecosystem, it is necessary to comprehensively consider multiple factors, such as its genetic background, planting environment and the presence of transferred or modified genes. Overall, the results suggest that transgenic maize 2A-7 expressing mCry1Ab and mCry2Ab proteins do not have significant impacts on soil physicochemical properties, enzyme activities and microbial functional diversity.

## Figures and Tables

**Table 1:** Changes in the physical and chemical properties of the rhizosphere soils of transgenic maize 2A-7 and non-transgenic maize at different developmental stages.

Developmental Stage	Variety	pH	Total Nitrogen (%)	Total Phosphorus (%)	Organic Matter (g/Kg)	Available Phosphorus (mg/Kg)	Alkali-Hydrolyzed Nitrogen (mg/Kg)
Seedling stage	2A-7	6.70 ± 0.10 ^a^	0.15 ± 0.01 ^a^	0.10 ± 0.01 ^a^	22.3 ± 2.70 ^a^	96.10 ± 4.62 ^a^	75.47 ± 5.67 ^a^
	CK	6.80 ± 0.00 ^a^	0.14 ± 0.01 ^a^	0.08 ± 0.03 ^a^	21.63 ± 1.34 ^a^	98.63 ± 10.73 ^a^	94.60 ± 16.32 ^a^
Jointing stage	2A-7	7.17 ± 0.06 ^a^	0.12 ± 0.01 ^a^	0.10 ± 0.02 ^a^	21.00 ± 4.66 ^a^	90.83 ± 3.50 ^a^	89.13 ± 11.48 ^a^
	CK	7.27 ± 0.06 ^a^	0.13 ± 0.01 ^a^	0.10 ± 0.01 ^a^	23.23 ± 0.55 ^a^	88.70 ± 0.35 ^a^	82.20 ± 9.00 ^a^
Tasseling stage	2A-7	7.40 ± 0.10 ^a^	0.14 ± 0.01 ^a^	0.10 ± 0.01 ^a^	21.60 ± 0.61 ^a^	80.00 ± 8.15 ^a^	85.47 ± 8.59 ^a^
	CK	7.50 ± 0.00 ^a^	0.13 ± 0.01 ^a^	0.11 ± 0.00 ^a^	24.93 ± 0.74 ^a^	80.80 ± 2.05 ^a^	79.93 ± 11.22 ^a^
Silking stage	2A-7	7.47 ± 0.06 ^a^	0.14 ± 0.02 ^a^	0.08 ± 0.03 ^a^	26.60 ± 6.32 ^a^	73.47 ± 14.72 ^a^	91.27 ± 17.07 ^a^
	CK	7.60 ± 0.00 ^a^	0.12 ± 0.02 ^a^	0.11 ± 0.00 ^a^	17.57 ± 0.32 ^a^	57.27 ± 1.80 ^a^	88.30 ± 16.63 ^a^
Full ripe stage	2A-7	7.73 ± 0.06 ^a^	0.13 ± 0.00 ^a^	0.12 ± 0.00 ^a^	20.60 ± 3.21 ^a^	59.33 ± 2.40 ^a^	85.10 ± 1.39 ^a^
	CK	7.80 ± 0.00 ^a^	0.12 ± 0.01 ^a^	0.12 ± 0.01 ^a^	17.17 ± 3.27 ^a^	59.17 ± 4.28 ^a^	85.63 ± 5.59 ^a^

The data have been shown as mean ± SE. Letters following the data indicate significant differences between the rhizosphere soil samples of transgenic maize 2A-7 and its non-transgenic control during different developmental stages (Student’s *t*-test was employed, *p* < 0.05). 2A-7 represents the transgenic maize 2A-7, and CK represents the non-transgenic control Dongdan 6531.

**Table 2:** Comparison of metabolic functional diversity indices of microbial communities in rhizosphere soils of transgenic maize 2A-7 and its non-transgenic control during different developmental periods.

Period	Variety	Shannon Index (H′)	Simpson Index (D)	McIntosh Index (U)
Seedling stage	2A-7	3.34 ± 0.00 ^a^	0.96 ± 0.00 ^a^	6.40 ± 0.20 ^a^
	CK	3.35 ± 0.01 ^a^	0.96 ± 0.00 ^a^	6.65 ± 0.21 ^a^
Jointing stage	2A-7	3.34 ± 0.01 ^a^	0.96 ± 0.00 ^a^	6.33 ± 0.22 ^a^
	CK	3.32 ± 0.02 ^a^	0.96 ± 0.00 ^a^	6.20 ± 0.52 ^a^
Tasseling stage	2A-7	3.34 ± 0.03 ^a^	0.96 ± 0.00 ^a^	6.57 ± 0.21 ^a^
	CK	3.34 ± 0.04 ^a^	0.96 ± 0.00 ^a^	6.35 ± 0.34 ^a^
Silking stage	2A-7	3.34 ± 0.00 ^a^	0.96 ± 0.00 ^a^	6.71 ± 0.10 ^a^
	CK	3.31 ± 0.01 ^a^	0.96 ± 0.00 ^a^	6.25 ± 0.27 ^a^
Full ripe stage	2A-7	3.33 ± 0.01 ^a^	0.96 ± 0.00 ^a^	5.91 ± 0.14 ^a^
	CK	3.33 ± 0.01 ^a^	0.96 ± 0.00 ^a^	5.92 ± 0.17 ^a^

The data have been shown as mean ± SE. Letters following the data indicate significant differences between the rhizosphere soil samples of transgenic maize 2A-7 and its non-transgenic control during different developmental stages (Student’s *t*-test was employed, *p* < 0.05). 2A-7 represents the transgenic maize 2A-7, and CK represents the non-transgenic control Dongdan 6531.

## Data Availability

The data presented in this study are available in the graphs and tables provided in the manuscript.
